# Development and validation of a tool to assess knowledge and attitudes towards generic medicines among students in Greece: The ATtitude TOwards GENerics (ATTOGEN) questionnaire

**DOI:** 10.1371/journal.pone.0188484

**Published:** 2017-11-29

**Authors:** Philip J. Domeyer, Vassilis Aletras, Fotios Anagnostopoulos, Vasiliki Katsari, Dimitris Niakas

**Affiliations:** 1 School of Social Sciences, Hellenic Open University, Patras, Greece; 2 Department of Business Administration, University of Macedonia, Thessaloniki, Greece; 3 Panteion University of Social and Political Sciences, Kallithea, Athens, Greece; 4 Department of Social and Educational Policy, University of Peloponnese, Korinthos, Greece; 5 Μedical School, National and Kapodistrian University of Athens, Athens, Greece; Aligarh Muslim University, INDIA

## Abstract

**Introduction:**

The use of generic medicines is a cost-effective policy, often dictated by fiscal restraints. To our knowledge, no fully validated tool exploring the students’ knowledge and attitudes towards generic medicines exists. The aim of our study was to develop and validate a questionnaire exploring the knowledge and attitudes of M.Sc. in Health Care Management students and recent alumni’s towards generic drugs in Greece.

**Materials and methods:**

The development of the questionnaire was a result of literature review and pilot-testing of its preliminary versions to researchers and students. The final version of the questionnaire contains 18 items measuring the respondents’ knowledge and attitude towards generic medicines on a 5-point Likert scale. Given the ordinal nature of the data, ordinal alpha and polychoric correlations were computed. The sample was randomly split into two halves. Exploratory factor analysis, performed in the first sample, was used for the creation of multi-item scales. Confirmatory factor analysis and Generalized Linear Latent and Mixed Model analysis (GLLAMM) with the use of the rating scale model were used in the second sample to assess goodness of fit. An assessment of internal consistency reliability, test-retest reliability, and construct validity was also performed.

**Results:**

Among 1402 persons contacted, 986 persons completed our questionnaire (response rate = 70.3%). Overall Cronbach’s alpha was 0.871. The conjoint use of exploratory and confirmatory factor analysis resulted in a six-scale model, which seemed to fit the data well. Five of the six scales, namely trust, drug quality, state audit, fiscal impact and drug substitution were found to be valid and reliable, while the knowledge scale suffered only from low inter-scale correlations and a ceiling effect. However, the subsequent confirmatory factor and GLLAMM analyses indicated a good fit of the model to the data.

**Conclusions:**

The ATTOGEN instrument proved to be a reliable and valid tool, suitable for assessing students’ knowledge and attitudes towards generic medicines.

## Introduction

Generic drugs are a means of cost-effective medical treatment favoring both patients and the healthcare system. Doctors are often urged to prescribe generic drugs as far as possible [1], although multiple strategies are necessary to change doctor prescribing habits [2]. The global deterioration of the economic situation and demographic change makes this urge more prominent nowadays, given the increased healthcare systems economic burden along with the imposed economic restraints. In particular, Greece is a country severely hit by the economic crisis. The imperative need for fiscal reform has led to fiscal austerity and budgetary cuts in many sectors, including healthcare expenditure. Strikingly, the pharmaceutical spending during the years 2010, 2011 and 2012 was sharply reduced (annual decrease of 10.1%, 9.0% and 18.4% respectively) and dropped from the 2009 level of 5.6billion euros to 2.37billion in 2013 [3,4]. In order to achieve those targets, policies have been implemented leading to drug expenditure rationalization and the introduction of a recommended list of medicines focusing on generic drugs.

However, despite the obvious cost-effectiveness of generic drugs considerable mistrust has been noted worldwide regarding their safety and efficacy and several studies indicate difficulties associated with treatment adherence or compliance [5–10]. According to Keenum et al [11], although generic medications are considered less expensive and of better value than their counterparts, fewer than 50% of the prescribers chose generic drugs for themselves. It seems that respondents’ attitudes towards generics are shaped from personal beliefs and knowledge–the latter being dissipated from mass media, friends and family-, previous experience with generics and trust to the prescriber [12].

Healthcare students are the future prescribers, dispensers and patients' advisors regarding medications, which makes them an important study target. Several studies have been published regarding their knowledge and perceptions regarding generic medicines [13–21]. Three of the abovementioned studies [17, 20, 21] are based on the only available validated questionnaire, although no validation study has been published. The use of such a validated tool would definitely increase the reliability and validity of studies on this important issue and would allow the direct comparability of studies. To our knowledge, only one study by Figueiras et al [22], in which a generic medicines scale was also created, was recently validated among the Malaysian population [23]. However, the scale underwent partial validation with the use of exploratory factor analysis only, without the use of any Structural Equation Modeling (SEM) technique.

To our knowledge, no study has emphasized on students and alumni of M.Sc. programs of Health Care Management, which are future health care leaders and pharmaceutical policy shapers. The future market share of generics will largely depend on the policy makers’ strategy, which is in turn influenced by their knowledge and attitude towards generics. The aim of our study was to develop and validate a questionnaire exploring the knowledge and attitudes of M.Sc. in Health Care Management students and that of recent alumni’s towards generic drugs.

## Materials and methods

### Study sample

The study sample comprised of all students and recent (up to 1 year from graduation) alumni of the M.Sc. program of Health Care Management at the Hellenic Open University. The Hellenic Open University provides distance education in both undergraduate and postgraduate levels. Contrary to the majority of other Public Universities, the students should pay fees for the cost of their studies. Students are selected without exams, by a public electronic lottery. Priority is given to candidates being at least 23 years old. For the abovementioned reasons, most students are usually older, with mean age being 32 years [24] and employed.

### Instrument creation

The MEDLINE, EMBASE, Cochrane and CINAHL databases were scanned for full articles published between January 2000 and November 2013, regarding knowledge/attitudes/perceptions/behaviours/opinions/views of doctors, pharmacists, consumers, patients and students towards generic drug use. Only full original research papers written in English were included. Title and abstract fields were searched for publications containing the terms: generic; knowledge; attitude; perception; behaviour; opinion; view; healthcare professional; physicians; doctors; pharmacists; consumers; patients; students. Boolean operators (AND/OR) were used to combine search components and to yield as many search results as possible. MeSH (Medical Subject Headings) terms were used where possible.

The databases' search returned a total of 1446 articles, the majority of which (873) related to consumers/patients/students; the PubMed searches proved the most successful, returning 972 articles. After the removal of duplicate findings, the reading of titles and abstracts of the remaining articles resulted in excluding those whose content was not relevant to this study. The identification of such articles was due to the design of the searches, using the selected search terms, in a deliberate attempt to ‘capture’ as many published articles in the field as possible. As a result, 60 articles were retained, eleven of which were not in English. Therefore, 49 papers [5–10, 11, 13, 14, 22, 25–63] were reviewed for the design of the study's questionnaire. Questions related to the aims of our study that commonly appeared in these studies and questions that yielded statistically significant differences among groups were retained. Accordingly, items were created and carefully phrased in order to avoid ambiguity, biases, double-barreled and double negative questions. Five sociodemographic questions regarding age, sex, marital and professional status and profession were added.

A preliminary version of the instrument was handed over to 10 researchers and experts in the fields of generics, attitude measurements and questionnaire construction. Face to face interviews were conducted by two of the researchers and recorded, during which they were asked to complete the questionnaire and highlight ambiguous or problematic items. They were also asked to propose additional items regarding aspects of a person’s attitude towards generic medicines not already captured by the instrument and to delete any trivial items, thus resulting in enhanced content validity and reliability. All necessary modifications, based on divergence of opinions between experts, were made to the instrument, which was subsequently handed over to 20 subjects randomly selected from the students’ and alumni pool. Again the same procedure was followed, which resulted in only minor textual changes and in the final version of the instrument to be handed over. This final version of the ATtitude TOwards GENerics (ATTOGEN) questionnaire consisted of close-ended Likert-type questions, apart from sociodemographic questions, with possible answers ranging from strongly agree (= 1) to strongly disagree (= 5).

### Instrument administration

The study took place between March and August 2014. The instrument was uploaded to Google Drive (Google Inc, California, USA). A web link was sent to all participants, who were asked to complete the questionnaire. In order to avoid missing data, all fields were marked as required and the questionnaire could not be submitted unless all questions had been answered. Monthly reminders were sent from the M.Sc. Director to the participants, in order to maximize the response rate.

### Ethics

Approval of the study by the Review Board of the Hellenic Open University was granted. Participants were reassured that their anonymity would be respected. Although the importance of achieving a high response rate was highlighted, participants were free to decide whether to complete the questionnaire or not. Furthermore, it was made clear that no recriminations would occur irrespective of their decision. There was no compensation for the participants. All participants provided their written informed consent to participate in this study. The informed consent form was sent electronically to the participants, who signed it and returned it by email or fax.

### Statistical analysis

The instrument created was psychometrically tested and subscales were created with the use of exploratory factor analysis. Subsequently, confirmatory factor analysis and generalized linear latent and mixed models were used to test the fit of the models to our data.

#### Exploratory factor analysis

To begin, the sample was randomly split into two halves. In the first sample, the exploratory factor analysis (EFA) was used for the creation of multi-item scales. The first step towards this direction was to check our data for normality, since factor analysis requires a multivariate normal distribution of the data. Although some degree of skewness was noted to our data, for large samples, like in our study, the multivariate Central Limit Theorem can be relied upon for a good approximation of normality, thus enabling the use of factor analysis [64]. Furthermore, we inspected the polychoric correlation matrix, in order to check whether our data were suitable for exploratory factor analysis.

Cronbach’s alpha coefficient was used to test for internal consistency [65]. Polychoric correlations were used since responses were expressed on a Likert (ordinal) scale [66,67]. The polychoric correlation matrix was created and ordinal versions of the alpha coefficients were also calculated. We also calculated Spearman’s coefficient, as an additional tool for the assessment of inter-item correlations, which is useful for ordinal data and robust to outliers, causing potential skewness.

We subsequently run the Bartlett’s test for sphericity to test the appropriateness of the factor model. We also calculated the Kaiser-Meyer-Olkin measure of Sampling Adequacy, which tests whether the partial correlations among variables are small; levels more than 0.5 are necessary for a satisfactory factor analysis to proceed [68], whereas values between 0.8 and 0.9 are considered great [69].

The exploratory factor analysis was carried out in the first half of the sample with the use of the polychoric correlation matrix. In order to further minimize the effect of non-normally distributed data, the principal axis factoring method was chosen. Following other research work, we also performed a typical factor analysis based on Pearson correlations, for comparative reasons, since the Likert scale could be treated as an interval or ratio scale [69]. The promax rotation was initially performed, assuming that the factors are correlated. In order to decide on the number of factors to retain, we used the Kaiser’s criterion, according to which all factors with eigenvalues<1 are dropped and also assessed the scree plot, as well as the results of the confirmatory factor analysis that followed [69]. Given that the Kaiser’s criterion is known for its tendency to overextract factors [70], we ultimately decided to adopt a less stringent approach, in order to reach an informative but relatively parsimonious model. The rotated factor loadings were computed; the commonly used threshold value of 0.5 was adopted for factor extraction [71,72]. The factor correlation matrix was calculated. According to Tabachnick & Fidell (2007) [73], if the absolute value of correlations exceed 0.32, then there is 10% (or more) overlap in variance among factors, enough variance to warrant oblique rotation. Finally, the predicted scoring coefficients were calculated and Cronbach’s alpha coefficients were computed for each factor separately and assessed according to the literature’s rule of thumb [74].

#### Structural equation modeling technique

Data from the second sample were used to implement the SEM technique, in order to test the fit of the models to our data. The confirmatory factor analysis and the Generalized Linear Latent and Mixed Model (GLLAMM) analysis were both used, as each method adopts a different statistical approach.

#### Confirmatory factor analysis

During the confirmatory factor analysis, we tried to assess the fit of the data to the model. Adjustments of the items contained in each factor were made based on post-estimation absolute and relative fit indices. Fit indices which were assessed included: i) the chi-squared test [75], ii) the Root Mean Square Error of Approximation (RMSEA), whose values of 0.06 or less are indicative of an acceptable model fit [76], iii) the Root Mean Square Residual (RMSR), whose values of 0.08 or less are indicative of an acceptable model fit [77] and iv) the Tucker Lewis Index (TLI) and the Comparative Fit Index (CFI), whose values of 0.9 or more are indicative of an acceptable model fit, with values of 0.95 and over being considered as a much better fit [78,79]. The squared multiple correlations (R^2^) for each observed variable of the model were also calculated [80].

#### Test-retest reliability

The sensitivity of the scale to external factors was assessed by examining test-retest reliability. The test-retest reliability was assessed using polychoric correlation coefficients, which were calculated for each subscale.

#### Construct validity

Multi-trait analysis was used to assess the construct validity of our tool; convergent and discriminant validity were therefore examined. Convergent validity refers to the degree to which two construct measures that theoretically should be related are truly related. Therefore, the item scale correlation between an item and the scale in which it allegedly belongs to should be expected to be higher than its correlations with irrelevant scales. According to Fornell and Lacker [81], average variance extracted (AVE) estimates should be 0.50 or above. On the other hand, discriminant validity refers to the degree to which two construct measures that theoretically should be unrelated are truly unrelated. In order that evidence for discriminant validity exists, the shared variances between paired constructs should be lower than the AVE of the individual constructs. Moreover, item-scale and inter-scale correlations were calculated. Due to the ordinal nature of the data, we computed the Pearson, Spearman and polychoric correlations, corrected for overlap, as per our previously published work [82].

#### Ceiling and floor effects

Furthermore, ceiling and floor effects were examined by each created subscale. The presence of ceiling and floor effects compromises the content validity of the scale. In particular, the ceiling and floor effects are encountered when several subjects’ responses consistently fall into the highest and lowest category, respectively.

#### Generalized linear latent and mixed model analysis

In the case of the GLLAMM analysis, two models, the partial credit model (PCM) [83] and the rating scale model (RSM) [84] were tested for each factor separately. The PCM is an extension of the Rasch model to polytomous items with ordered response categories. The RSM is a special case of the PCM, used when response categories have the same meaning for all items and under the assumption that the differences in the step difficulties for different categories are the same for all items [85]. In other words, the response scale is fixed for all items, i.e. the response threshold parameters are assumed to be identical across items.

To compare the various models’ output in both confirmatory factor analysis and GLLAMM analysis, a likelihood ratio test was performed. Finally, estimates and predictions were made based on the chosen GLLAMM model.

Statistical analysis was performed using Stata version 13.1 (Stata Corp., Texas, USA). Two-tailed p values <0.05 were considered statistically significant.

## Results

Among 1402 persons contacted, 986 persons completed the questionnaire (response rate = 70.3%). The mean age of the participants was 39.8±7.11 years. The sociodemographic characteristics of our study sample are presented in Table 1.

**Table 1:** Sociodemographic characteristics of the study sample.

Variables	N (%)
*Sex*	
Male	366 (37.1%)
Female	620 (62.9%)
*Marrital status*	
Single	313 (31.7%)
Married	628 (63.7%)
Divorced	42 (4.3%)
Widowed	3 (0.3%)
*Professional status*	
Employed	938 (95.1%)
Unemployed	48 (4.9%)
*Profession*	
Doctor	270 (27.4%)
Dentist	32 (3.2%)
Pharmacist	36 (3.6%)
Nurse	194 (19.7%)
Other health professional	266 (27.0%)
Other profession	188 (19.1%)
*Educational status*	
Students	716 (72.6%)
Graduates	270 (27.4%)

The questionnaire that was handed over to the participants of our study comprised 24 items, apart from the sociodemographic questions. Of the 24 items, 2 items (“I know if my current medications include generic medicines”) and (“I have noticed substantial differences between brand name and generic medicines”) were related to the intake of medications and were excluded since the majority of the participants (665/986, 67.4%) were not under medications. On inspecting the polychoric correlation matrix, two variables (“I am satisfied with the information I get regarding generic medicines” and “Among two generic medicines, I would trust more the one that would be manufactured in Greece”) were found to have low correlation coefficients with the rest of the variables, were considered unsuitable for factor analysis and were hence excluded. The remaining items of the questionnaire that underwent statistical analysis, along with their descriptive statistics, are presented in Table 2.

**Table 2:** Descriptive statistics of the items.

Item	Description	Mean±SD	Median (IQR)
**1**	I know what generic medications are	1.502±0.795	1 (1)
**2**	I know the difference between generics and brand name medications	1.526±0.771	1 (1)
**3**	A brand name and a generic medication contain the same active substance	1.583±0.903	1 (1)
**4**	The potency of generic and brand name medications is the same	2.466±1.125	2 (1)
**5**	The safety of generic and brand name medications is the same	2.756±1.109	3 (2)
**6**	The production standards of generic and brand name medications are the same	2.903±1.124	3 (2)
**7**	The price of generic medications is considerably lower than brand name medications	1.843±0.975	2 (1)
**8**	Substitution of brand-name with generic medicines can also be done by pharmacists	3.516±1.368	4 (3)
**9**	Substitution of brand-name with generic medicines should only be done by doctors	1.861±1.130	1 (1)
**10**	I believe that the use of generic medicines will reduce any relationships between doctors and pharmaceutical companies against the rules	2.848±1.298	3 (2)
**11**	I believe that the use of generic medicines will reduce the total cost of therapy	2.205±1.061	2 (2)
**12**	I would trust more a brand name than a generic medicine	2.258±1.105	2 (2)
**13**	I would trust more a doctor who would prescribe me a brand-name rather than a generic medicine	3.128±1.134	3 (2)
**14**	I am skeptical about generic medicines because of their lower price	3.647±1.074	4 (1)
**15**	I believe that generics were invented and promoted in order to resolve the financial crisis of social security institutions at the expense of citizens	3.267±1.251	3 (2)
**16**	Generic medicines have more undesirable effects (side-effects) than brand name medicines	3.215±1.107	3 (1)
**17**	The Greek authorities are able to detect possible irregularities in the production of generic medicines	3.402±1.139	4 (1)
**18**	The Greek authorities are able to detect in time and retract batches of generic drugs with reduced potency and/or safety	3.427±1.113	4 (1)
**19**	In case of ineffectiveness of Greek authorities, European authorities are capable of detecting possible irregularities in potency and/or safety of generic medicines in the Greek market	2.924±1.054	3 (2)
**20**	I would be worried if my medication was changed from brand-name to generic	2.907±1.204	3 (2)

^a^ IQR, Interquartile range

### Exploratory factor analysis

Cronbach’s alpha and ordinal Cronbach’s alpha coefficients for the entire questionnaire were both high and did not differ substantially (0.871 and 0.893 respectively). The Bartlett’s test for sphericity was statistically significant (p<0.0001) and the Kaiser-Meyer-Olkin measure of sampling adequacy was 0.858. The factor analysis based on polychoric correlations with subsequent promax rotation yielded 6 factors, which account for 96.1% of the total variance and are shown in Table 3. Due to negative factor loadings, item 9 was inversely recoded. The factor correlation matrix was also calculated (Table 4). Most correlations exceeded 0.32; therefore, oblique rotation was performed.

**Table 3:** Factor analysis with polychoric correlations (rotated factor loadings^a^).

Item	Factor 1	Factor 2	Factor 3	Factor 4	Factor 5	Factor 6
**1**	0.016	-0.045	0.031	0.006	**0.925**	0.017
**2**	0.009	-0.046	-0.065	0.105	**0.927**	-0.023
**3**	-0.021	0.408	-0.033	-0.083	**0.510**	0.104
**4**	-0.097	**0.745**	-0.076	0.125	-0.019	-0.033
**5**	-0.098	**0.878**	0.050	-0.056	-0.016	0.0002
**6**	-0.068	**0.702**	0.129	0.052	0.019	-0.017
**7**	0.113	0.105	0.055	**0.570**	0.201	-0.078
**8**	0.105	0.049	0.071	0.062	-0.002	**0.836**
**9**^**b**^	-0.093	-0.071	-0.042	-0.023	0.036	**0.857**
**10**	-0.124	0.019	0.011	**0.588**	-0.180	0.193
**11**	-0.131	0.080	-0.020	**0.695**	0.095	-0.024
**12**	**0.661**	-0.278	0.031	-0.042	0.130	0.026
**13**	**0.825**	0.027	0.040	-0.108	0.084	0.006
**14**	**0.623**	0.099	-0.083	0.099	-0.262	0.011
**15**	**0.621**	-0.022	-0.020	0.152	-0.154	-0.047
**16**	**0.625**	-0.300	0.036	0.018	0.064	-0.020
**17**	0.042	0.017	**0.932**	0.012	0.018	-0.024
**18**	0.018	0.023	**0.948**	-0.045	-0.057	0.052
**19**	-0.118	-0.021	**0.567**	0.091	0.004	-0.043
**20**	**0.775**	-0.057	-0.006	-0.139	-0.055	0.010

^a^ Numbers in bold indicate the highest factor loadings for each item.

^b^ Due to negative factor loading, item was inversely recoded

**Table 4:** Factors correlation matrix in exploratory factor analysis with oblique rotation.

Factor	1	2	3	4	5	6
**1**	1					
**2**	-0.614	1				
**3**	-0.318	0.348	1			
**4**	-0.301	0.527	0.463	1		
**5**	-0.378	0.399	-0.021	0.006	1	
**6**	-0.380	0.252	0.186	0.240	0.091	1

### Confirmatory factor analysis

A confirmatory factor analysis was subsequently run to explore the strength of the proposed 20-item scale, which yielded moderate fit results (RMSEA = 0.061, RMSR = 0.072, TLI = 0.912, CFI = 0.928). The 6-scale approach seemed to have the best fit. The increase of factors retained from 6 to 7 (matching the scree plot results) or the decrease to 5 or 4 factors (nearly matching or matching the results of the Kaiser’s criterion) provided even worse fit results at the subsequent confirmatory factor analysis. After examining the exclusion of item(s) with the weakest factor loadings and the highest uniqueness scores on the 6 factor model, it was found that deleting items 14 and 15, thus reducing the increased number in scale 1 from 6 to 4, would provide a better fit (RMSEA = 0.059, RMSR = 0.063, TLI = 0.927, CFI = 0.942). In an attempt to further ameliorate the model fit, the free estimation of the covariance between the error terms of items 1 and 2 as well as items 17 and 18 was allowed. These specific pairs of items were chosen, because the variables within each pair are measuring similar attitudes and have very strong correlation with each other. The resulting model provided the best fit (RMSEA = 0.050, RMSR = 0.048, TLI = 0.948, CFI = 0.960). Indeed, this newly introduced correlation was statistically significant according to the likelihood ratio test (p<0.0001). Table 5 presents the squared multiple correlations (R^2^) for each observed variable of the model. Most of them ranged from acceptable to good, apart from items 1, 2 and 7. By introducing the correlated error term for items 1 and 2, the R^2^ of those variables decreased. This is fairly rational, since the covariance between these two items no longer contributes to explaining the factor “knowledge”, given that the covariance between the error terms δ_1_ and δ_2_ is now freely estimated, as suggested by Kolenikov.[86] Regarding variable 7, its low squared multiple correlation is consistent with its comparatively lower factor loading in explanatory factor analysis.

**Table 5:** Squared multiple correlations (R^2^) for each observed variable of the model.

Scale/item	R^2^
*Trust*	
12	0.468
13	0.406
16	0.566
20	0.711
*Drug quality*	
4	0.444
5	0.737
6	0.585
*State audit*	
17	0.741
18	0.741
19	0.417
*Fiscal impact*	
7	0.248
10	0.415
11	0.584
*Knowledge*	
1	0.219
2	0.278
3	0.792
*Drug substitution*	
8	0.491
9	0.672

The Cronbach’s alpha coefficients were calculated for each generated scale andwere found to be good for scales 1, 2 and 3 (0.821, 0.805 and 0.847 respectively), acceptable for scales 5 and 6 (0.777 and 0.719) and questionable for scale 4 (0.655). Table 6 presents the internal consistency reliability of the 6 scales generated from the factor analysis, including Cronbach’s alpha coefficients, mean and median values.

**Table 6:** Internal consistency reliability and scale scores.

Item	Scale/item description	Cronbach’s Alpha	Mean±SD score	Median (IQR)^a^ score
	*Scale 1*: *TRUST*	0.821^b^	2.83±0.896	2.75 (1.250)
**12**	“I would trust more a brand name than a generic medicine”	0.876	2.18±1.082	2 (2)
**13**	“I would trust more a doctor who would prescribe me a brand-name rather than a generic medicine”	0.794	3.06±1.114	3 (2)
**16**	“Generic medicines have more undesirable effects (side-effects) than brand name medicines”	0.766	3.20±1.074	3 (1)
**20**	“I would be worried if my medication was changed from brand-name to generic”	0.885	2.88±1.153	3 (2)
	*Scale 2*: *Drug quality*	0.805^b^	2.71±0.938	2.67 (1.333)
**4**	“The potency of generic and brand name medications is the same”	0.843	2.45±1.104	2 (1)
**5**	“The safety of generic and brand name medications is the same”	0.898	2.74±1.096	3 (2)
**6**	“The production standards of generic and brand name medications are the same”	0.915	2.94±1.116	3 (2)
	*Scale 3*: *State audit*	0.847^b^	3.19±0.993	3.33 (1.333)
**17**	“The Greek authorities are able to detect possible irregularities in the production of generic medicines”	0.956	3.35±1.165	4 (1)
**18**	“The Greek authorities are able to detect in time and retract batches of generic drugs with reduced potency and/or safety”	0.958	3.35±1.141	4 (1)
**19**	“In case of ineffectiveness of Greek authorities, European authorities are capable of detecting possible irregularities in potency and/or safety of generic medicines in the Greek market.	0.880	2.88±1.064	3 (2)
	*Scale 4*: *Fiscal impact*	0.655^b^	2.29±0.855	2.33 (1)
**7**	“The price of generic medications is considerably lower than brand name medications”	0.814	1.86±1.018	2 (1)
**10**	“I believe that the use of generic medicines will reduce any relationships between doctors and pharmaceutical companies against the rules”	0.855	2.83±1.251	3 (2)
**11**	“I believe that the use of generic medicines will reduce the total cost of therapy”	0.886	2.18±1.051	2 (2)
	*Scale 5*: *Knowledge*	0.777^b^	1.57±0.712	1.33 (1)
**1**	“I know what generic medications are”	0.919	1.53±0.822	1 (1)
**2**	“I know the difference between generics and brand name medications”	0.934	1.53±0.777	1 (1)
**3**	“A brand name and a generic medication contain the same active substance”	0.855	1.65±0.959	1 (1)
	*Scale 6*: *Drug substitution*	0.719^b^	3.89±1.079	4 (2)
**8**	“Substitution of brand-name with generic medicines can also be done by pharmacists”	0.942	3.54±1.348	4 (3)
**9**	“Substitution of brand-name with generic medicines should only be done by doctors”	0.924	4.23±1.080	5 (1)

^a^ IQR, Interquartile range

^b^ Cronbach’s alpha for summary scale

### Test-retest reliability

Thirty randomly selected participants were contacted for a second completion of the questionnaire, which took place two weeks after the initial administration; among them twenty eight completed it (response rate 93.3%). The test-retest reliability results were also good, with polychoric correlation coefficients all above 0.80.

### Construct validity

The results of the multi-trait analysis using polychoric correlations are shown in Table 7. Pearson and Spearman correlations were also calculated, with similar results. Inter-scale correlations were calculated and all of them were found highly statistically significant (p<0.0001). An exception was noted for the “Knowledge” scale, regarding inter-scale correlations with “State audit”(p = 0.199), “Fiscal impact” (p = 0.183) and “Drug substitution” (p = 0.866) scales. The AVE estimates were computed and were found to be above 0.5, except for scale 4 (0.496), and higher than the corresponding estimated squared correlations. Therefore, there was adequate overall evidence for convergent and discriminatory validity, respectively.

**Table 7:** Multi-trait analysis with polychoric correlations^a^.

Scale/item	Item-Scale Correlations					
	Trust	Drug quality	State audit	Fiscal impact	Knowledge	Drug substitution
***Trust***						
**12**	0.671	-0.516	-0.227	-0.279	-0.116	-0.199
**13**	0.626	-0.384	-0.176	-0.272	-0.174	-0.156
**16**	0.661	-0.533	-0.198	-0.313	-0.226	-0.182
**20**	0.743	-0.591	-0.266	-0.307	-0.263	-0.216
***Drug quality***						
**4**	-0.481	0.634	0.172	0.267	0.352	0.184
**5**	-0.611	0.753	0.374	0.344	0.356	0.219
**6**	-0.500	0.711	0.419	0.392	0.274	0.186
***State audit***						
**17**	-0.283	0.365	0.864	0.363	0.086	0.191
**18**	-0.267	0.356	0.870	0.336	0.062	0.146
**19**	-0.168	0.266	0.632	0.297	0.075	0.130
***Fiscal impact***						
**7**	-0.191	0.284	0.291	0.443	0.124	0.100
**10**	-0.313	0.310	0.292	0.520	0.005	0.269
**11**	-0.353	0.347	0.305	0.623	0.168	0.202
***Knowledge***						
**1**	-0.184	0.256	0.067	0.039	0.771	-0.003
**2**	-0.178	0.274	0.064	-0.011	0.822	-0.001
**3**	-0.305	0.481	0.066	0.141	0.612	0.046
***Drug substitution***						
**8**	-0.153	0.208	0.198	0.252	0.061	0.680
**9**	-0.283	0.238	0.090	0.199	0.041	0.680

^a^ Pearson and Spearman correlations were also calculated, with similar outcomes (results not shown)

### Ceiling and floor effects

Regarding ceiling effects, the percentages of observations falling into the best attitude score category for the six summated scales were 2.03%, 6.29%, 2.84%, 9.94%, 44.62% and 2.43%. The respective percentages for the flooring effects were 1.83%, 2.43%, 4.87%, 0.41%, 0.20% and 19.41%. Only the “Knowledge” scale presented a ceiling effect.

### Generalized linear latent and mixed model analysis

The GLLAMM analysis was also performed on each scale separately. Both the partial credit model and the rating scale model were tested. Since the likelihood ratio test was highly insignificant in all scales (p>0.9) and the rating scale model is more parsimonious, the latter was chosen. According to this model, prediction estimates were calculated and category probability curves (CPCs) were created. S1–S3 Figs show CPCs for each of the three items of the “fiscal impact” scale, which indicate good model fit and no category disordering. Corresponding graphs were also created for the other scales, with similar results.

## Discussion

The ATTOGEN questionnaire was developed and validated in order to assess the knowledge and attitude of students towards generic medicines. The questionnaire initially contained 24 items. Although two questions related to the intake of medications were excluded, the content validity of our questionnaire does not seem to be significantly compromised because of the existence of items 1 and 2, which ask participants whether they know what generics are as well as the differences between brand name and generics. The combination of exploratory and confirmatory factor analysis revealed items with low correlation coefficients and items that substantially worsened the model fit, which were also excluded from the composite scales. The final instrument consisted of 6 scales, namely trust (4 items), drug quality (3 items), state audit (3 items), fiscal impact (3 items), knowledge (3 items) and drug substitution (2 items). Because the criteria regarding the number of factors to be retained during the exploratory factor analysis were discordant–Kaiser’s criterion suggested 4 factors and the scree plot 7 factors -, the “trial and error” approach was adopted, using confirmatory factor analysis to test the fit of various models. The choice of the final model was based on the goodness of fit of the models, while respecting the content validity of the questionnaire.

The ATTOGEN questionnaire was found to have good overall internal consistency. Regarding the psychometric properties of the scales, satisfactory internal consistency reliability, test-retest reliability and construct validity were noted, with the exceptions of scale 5 (knowledge) with low inter-scale correlations. A ceiling effect was only present in the knowledge scale, which is an effect that seems to appear quite commonly in the literature [77,87]. Further studies may be needed to examine a potential improvement or exclusion of the knowledge scale without jeopardizing content validity. Nevertheless, the confirmatory factor analysis and the GLLAMM analysis both indicated a good overall fit. We believe that the confirmation of this finding with the concomitant use of two different SEM techniques is particularly meaningful.

In the study by Figueiras et al [22], in which a generic medicines scale was also created and recently validated among the Malaysian population [23], the initial number of items was not too different (18 items vs 24 items in our study). However, the exploratory factor analysis led to two factors only, namely efficacy and similarity, instead of six factors in our study. Moreover, the fit of this model to the data was not assessed, which could have altered the final results.

The strengths of the present study include the creation of a validated questionnaire regarding generics, which fills an important gap in the international literature, with the use of a large number of participants who completed the questionnaire with no missing values. Another strength was the concomitant use of two SEM techniques, both confirming the goodness of fit of our results. Our study, however, bears certain limitations that should be addressed. Firstly, our sample may not be representative of Greek students in conventional universities; health management students may express different views compared to other students, because of their specific academic background and because the majority of them are already working in clinical practice as healthcare providers. On the other hand, students from the Hellenic Open University belong to various age, economic and sociodemographic groups, because of the nature of the study environment (distance learning), which may somewhat increase the generalizability of the study’s results. In addition, due to the lack of any other similar questionnaire translated in Greek, the convergent validity of the questionnaire was not fully assessed. Finally, the low inter-scale correlations and the ceiling effect noted in scale 5 (knowledge) may deteriorate the psychometric properties of the questionnaire.

## Conclusions

To conclude, the use of exploratory and confirmatory factor analysis as well as GLLAMM modeling technique revealed five reliable and valid multi-item scales: trust, drug quality, state audit, fiscal impact and drug substitution. A sixth scale (knowledge) was found to suffer from relatively low internal consistency reliability and low inter-scale correlations. However, both the confirmatory factor and the GLLAMM analyses indicated an adequate fit, suggesting that the uncompromised use of all six scales could be justified. Further studies should be elaborated in order to further test the questionnaire to large representative samples of the Greek population, in order to ascertain its generalizability.

## Supporting information

S1 Fig Category probability curves for item 7 under the rating scale model. (TIF)

S2 Fig Category probability curves for item 10 under the rating scale model. (TIF)

S3 Fig Category probability curves for item 11 under the rating scale model. (TIF)

S1 File Copy of generics—PLOS ONE. (DTA)

S1 Table Sociodemographic characteristics of the study sample. (DOCX)

S2 Table Descriptive statistics of the items. (DOCX)

S3 Table Factor analysis with polychoric correlations (rotated factor loadings). (DOCX)

S4 Table Factors correlation matrix in Exploratory Factor Analysis with oblique rotation. (DOCX)

S5 Table Squared multiple correlations (R^2^) for each observed variable of the model. (DOCX)

S6 Table Internal consistency reliability and scale scores. (DOCX)

S7 Table Multi-trait analysis with polychoric correlations. (DOCX)
