# Supplementary figures and images for: Development and validation of a tool to assess knowledge and attitudes towards generic medicines among students in Greece: The ATtitude TOwards GENerics (ATTOGEN) questionnaire
Source: PLoS One. 2017 Nov 29;12(11):e0188484. doi: 10.1371/journal.pone.0188484 (PMC5706728; doi:10.1371/journal.pone.0188484)

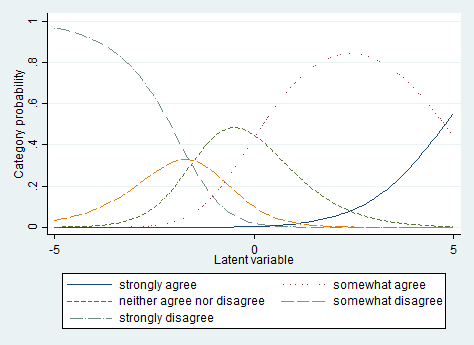

Supplement: S1 Fig — (TIF) [file pone.0188484.s001.tif]

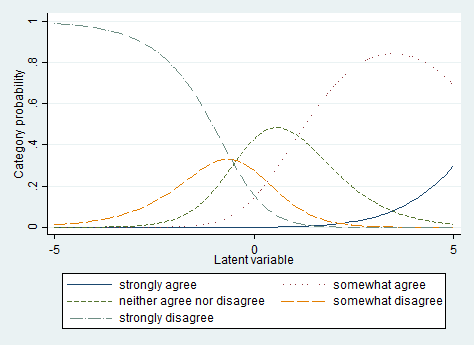

Supplement: S2 Fig — (TIF) [file pone.0188484.s002.tif]

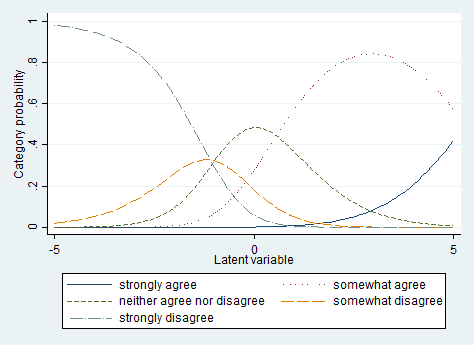

Supplement: S3 Fig — (TIF) [file pone.0188484.s003.tif]
