# Supplementary material for: Development and validation of a tool to assess knowledge and attitudes towards generic medicines among students in Greece: The ATtitude TOwards GENerics (ATTOGEN) questionnaire
Source: PLoS One. 2017 Nov 29;12(11):e0188484. doi: 10.1371/journal.pone.0188484 (PMC5706728; doi:10.1371/journal.pone.0188484)
Supplement: S1 Table — (DOCX) [file pone.0188484.s005.docx]

**Table 1. Sociodemographic characteristics of the study sample.**

| **Variables** | **N (%)** |
| --- | --- |
| *Sex* |  |
| Male | 366 (37.1%) |
| Female | 620 (62.9%) |
| *Marrital status* |  |
| Single | 313 (31.7%) |
| Married | 628 (63.7%) |
| Divorced | 42 (4.3%) |
| Widowed | 3 (0.3%) |
| *Professional status* |  |
| Employed | 938 (95.1%) |
| Unemployed | 48 (4.9%) |
| *Profession* |  |
| Doctor | 270 (27.4%) |
| Dentist | 32 (3.2%) |
| Pharmacist | 36 (3.6%) |
| Nurse | 194 (19.7%) |
| Other health professional | 266 (27.0%) |
| Other profession | 188 (19.1%) |
| *Educational status* |  |
| Students | 716 (72.6%) |
| Graduates | 270 (27.4%) |
