# Supplementary material for: Development and validation of a tool to assess knowledge and attitudes towards generic medicines among students in Greece: The ATtitude TOwards GENerics (ATTOGEN) questionnaire
Source: PLoS One. 2017 Nov 29;12(11):e0188484. doi: 10.1371/journal.pone.0188484 (PMC5706728; doi:10.1371/journal.pone.0188484)
Supplement: S2 Table — (DOCX) [file pone.0188484.s006.docx]

**Table 2. Descriptive statistics of the items.**

| **Item** | **Description** | **Mean±SD** | **Median (IQR)** |
| --- | --- | --- | --- |
| **1** | I know what generic medications are | 1.50±0.795 | 1 (1) |
| **2** | I know the difference between generics and brand name medications | 1.53±0.771 | 1 (1) |
| **3** | A brand name and a generic medication contain the same active substance | 1.58±0.903 | 1 (1) |
| **4** | The potency of generic and brand name medications is the same | 2.47±1.125 | 2 (1) |
| **5** | The safety of generic and brand name medications is the same | 2.76±1.109 | 3 (2) |
| **6** | The production standards of generic and brand name medications are the same | 2.90±1.124 | 3 (2) |
| **7** | The price of generic medications is considerably lower than brand name medications | 1.84±0.975 | 2 (1) |
| **8** | Substitution of brand-name with generic medicines can also be done by pharmacists | 3.52±1.368 | 4 (3) |
| **9** | Substitution of brand-name with generic medicines should only be done by doctors | 1.86±1.130 | 1 (1) |
| **10** | I believe that the use of generic medicines will reduce any relationships between doctors and pharmaceutical companies against the rules | 2.85±1.298 | 3 (2) |
| **11** | I believe that the use of generic medicines will reduce the total cost of therapy | 2.20±1.061 | 2 (2) |
| **12** | I would trust more a brand name than a generic medicine | 2.26±1.105 | 2 (2) |
| **13** | I would trust more a doctor who would prescribe me a brand-name rather than a generic medicine | 3.13±1.134 | 3 (2) |
| **14** | I am skeptical about generic medicines because of their lower price | 3.65±1.074 | 4 (1) |
| **15** | I believe that generics were invented and promoted in order to resolve the financial crisis of social security institutions at the expense of citizens | 3.27±1.251 | 3 (2) |
| **16** | Generic medicines have more undesirable effects (side-effects) than brand name medicines | 3.22±1.107 | 3 (1) |
| **17** | The Greek authorities are able to detect possible irregularities in the production of generic medicines | 3.40±1.139 | 4 (1) |
| **18** | The Greek authorities are able to detect in time and retract batches of generic drugs with reduced potency and/or safety | 3.43±1.113 | 4 (1) |
| **19** | In case of ineffectiveness of Greek authorities, European authorities are capable of detecting possible irregularities in potency and/or safety of generic medicines in the Greek market | 2.92±1.054 | 3 (2) |
| **20** | I would be worried if my medication was changed from brand-name to generic | 2.91±1.204 | 3 (2) |
