# Supplementary material for: Development and validation of a tool to assess knowledge and attitudes towards generic medicines among students in Greece: The ATtitude TOwards GENerics (ATTOGEN) questionnaire
Source: PLoS One. 2017 Nov 29;12(11):e0188484. doi: 10.1371/journal.pone.0188484 (PMC5706728; doi:10.1371/journal.pone.0188484)
Supplement: S3 Table — (DOCX) [file pone.0188484.s007.docx]

**Table 3. Factor analysis with polychoric correlations (rotated factor loadings^a^).**

| **Item** | **Factor 1** | **Factor 2** | **Factor 3** | **Factor 4** | **Factor 5** | **Factor 6** |
| --- | --- | --- | --- | --- | --- | --- |
| **1** | 0.016 | -0.045 | 0.031 | 0.006 | **0.925** | 0.017 |
| **2** | 0.009 | -0.046 | -0.065 | 0.105 | **0.927** | -0.023 |
| **3** | -0.021 | 0.408 | -0.033 | -0.083 | **0.510** | 0.104 |
| **4** | -0.097 | **0.745** | -0.076 | 0.125 | -0.019 | -0.033 |
| **5** | -0.098 | **0.878** | 0.050 | -0.056 | -0.016 | 0.0002 |
| **6** | -0.068 | **0.702** | 0.129 | 0.052 | 0.019 | -0.017 |
| **7** | 0.113 | 0.105 | 0.055 | **0.570** | 0.201 | -0.078 |
| **8** | 0.105 | 0.049 | 0.071 | 0.062 | -0.002 | **0.836** |
| **9^b^** | -0.093 | -0.071 | -0.042 | -0.023 | 0.036 | **0.857** |
| **10** | -0.124 | 0.019 | 0.011 | **0.588** | -0.180 | 0.193 |
| **11** | -0.131 | 0.080 | -0.020 | **0.695** | 0.095 | -0.024 |
| **12** | **0.661** | -0.278 | 0.031 | -0.042 | 0.130 | 0.026 |
| **13** | **0.825** | 0.027 | 0.040 | -0.108 | 0.084 | 0.006 |
| **14** | **0.623** | 0.099 | -0.083 | 0.099 | -0.262 | 0.011 |
| **15** | **0.621** | -0.022 | -0.020 | 0.152 | -0.154 | -0.047 |
| **16** | **0.625** | -0.300 | 0.036 | 0.018 | 0.064 | -0.020 |
| **17** | 0.042 | 0.017 | **0.932** | 0.012 | 0.018 | -0.024 |
| **18** | 0.018 | 0.023 | **0.948** | -0.045 | -0.057 | 0.052 |
| **19** | -0.118 | -0.021 | **0.567** | 0.091 | 0.004 | -0.043 |
| **20** | **0.775** | -0.057 | -0.006 | -0.139 | -0.055 | 0.010 |

^a^ Numbers in bold indicate the highest factor loadings for each item.
^b^ Due to negative factor loading, item was inversely recoded
