# Supplementary material for: Development and validation of a tool to assess knowledge and attitudes towards generic medicines among students in Greece: The ATtitude TOwards GENerics (ATTOGEN) questionnaire
Source: PLoS One. 2017 Nov 29;12(11):e0188484. doi: 10.1371/journal.pone.0188484 (PMC5706728; doi:10.1371/journal.pone.0188484)
Supplement: S4 Table — (DOCX) [file pone.0188484.s008.docx]

**Table 4. Factors correlation matrix in Exploratory Factor Analysis with oblique rotation.**

| **Factor** | **1** | **2** | **3** | **4** | **5** | **6** |
| --- | --- | --- | --- | --- | --- | --- |
| **1** | 1 |  |  |  |  |  |
| **2** | -0.614 | 1 |  |  |  |  |
| **3** | -0.318 | 0.348 | 1 |  |  |  |
| **4** | -0.301 | 0.527 | 0.463 | 1 |  |  |
| **5** | -0.378 | 0.399 | -0.021 | 0.006 | 1 |  |
| **6** | -0.380 | 0.252 | 0.186 | 0.240 | 0.091 | 1 |
