# Supplementary material for: Development and validation of a tool to assess knowledge and attitudes towards generic medicines among students in Greece: The ATtitude TOwards GENerics (ATTOGEN) questionnaire
Source: PLoS One. 2017 Nov 29;12(11):e0188484. doi: 10.1371/journal.pone.0188484 (PMC5706728; doi:10.1371/journal.pone.0188484)
Supplement: S5 Table — (DOCX) [file pone.0188484.s009.docx]

**Table 5. Squared multiple correlations (R^2^) for each observed variable of the model.**

| **Scale/item** | **R^2^** |
| --- | --- |
| *Trust* |  |
| 12 | 0.468 |
| 13 | 0.406 |
| 16 | 0.566 |
| 20 | 0.711 |
| *Drug quality* |  |
| 4 | 0.444 |
| 5 | 0.737 |
| 6 | 0.585 |
| *State audit* |  |
| 17 | 0.741 |
| 18 | 0.741 |
| 19 | 0.417 |
| *Fiscal impact* |  |
| 7 | 0.248 |
| 10 | 0.415 |
| 11 | 0.584 |
| *Knowledge* |  |
| 1 | 0.219 |
| 2 | 0.278 |
| 3 | 0.792 |
| *Drug substitution* |  |
| 8 | 0.491 |
| 9 | 0.672 |
