# Supplementary material for: Development and validation of a tool to assess knowledge and attitudes towards generic medicines among students in Greece: The ATtitude TOwards GENerics (ATTOGEN) questionnaire
Source: PLoS One. 2017 Nov 29;12(11):e0188484. doi: 10.1371/journal.pone.0188484 (PMC5706728; doi:10.1371/journal.pone.0188484)
Supplement: S6 Table — (DOCX) [file pone.0188484.s010.docx]

**Table 6. Internal consistency reliability and scale scores.**

| **Item** | **Scale/item description** | **Cronbach’s Alpha** | **Mean±SD score** | **Median (IQR)**^a^ **score** |
| --- | --- | --- | --- | --- |
|  | *Scale 1: TRUST* | 0.821^b^ | 2.83±0.896 | 2.75 (1.250) |
| **12** | “I would trust more a brand name than a generic medicine” | 0.876 | 2.18±1.082 | 2 (2) |
| **13** | “I would trust more a doctor who would prescribe me a brand-name rather than a generic medicine” | 0.794 | 3.06±1.114 | 3 (2) |
| **16** | “Generic medicines have more undesirable effects (side-effects) than brand name medicines” | 0.766 | 3.20±1.074 | 3 (1) |
| **20** | “I would be worried if my medication was changed from brand-name to generic” | 0.885 | 2.88±1.153 | 3 (2) |
|  | *Scale 2: Drug quality* | 0.805^b^ | 2.71±0.938 | 2.67 (1.333) |
| **4** | “The potency of generic and brand name medications is the same” | 0.843 | 2.45±1.104 | 2 (1) |
| **5** | “The safety of generic and brand name medications is the same” | 0.898 | 2.74±1.096 | 3 (2) |
| **6** | “The production standards of generic and brand name medications are the same” | 0.915 | 2.94±1.116 | 3 (2) |
|  | *Scale 3: State audit* | 0.847^b^ | 3.19±0.993 | 3.33 (1.333) |
| **17** | “The Greek authorities are able to detect possible irregularities in the production of generic medicines” | 0.956 | 3.35±1.165 | 4 (1) |
| **18** | “The Greek authorities are able to detect in time and retract batches of generic drugs with reduced potency and/or safety” | 0.958 | 3.35±1.141 | 4 (1) |
| **19** | “In case of ineffectiveness of Greek authorities, European authorities are capable of detecting possible irregularities in potency and/or safety of generic medicines in the Greek market. | 0.880 | 2.88±1.064 | 3 (2) |
|  | *Scale 4: Fiscal impact* | 0.655^b^ | 2.29±0.855 | 2.33 (1) |
| **7** | “The price of generic medications is considerably lower than brand name medications” | 0.814 | 1.86±1.018 | 2 (1) |
| **10** | “I believe that the use of generic medicines will reduce any relationships between doctors and pharmaceutical companies against the rules” | 0.855 | 2.83±1.251 | 3 (2) |
| **11** | “I believe that the use of generic medicines will reduce the total cost of therapy” | 0.886 | 2.18±1.051 | 2 (2) |
|  | *Scale 5: Knowledge* | 0.777^b^ | 1.57±0.712 | 1.33 (1) |
| **1** | “I know what generic medications are” | 0.919 | 1.53±0.822 | 1 (1) |
| **2** | “I know the difference between generics and brand name medications” | 0.934 | 1.53±0.777 | 1 (1) |
| **3** | “A brand name and a generic medication contain the same active substance” | 0.855 | 1.65±0.959 | 1 (1) |
|  | *Scale 6: Drug substitution* | 0.719^b^ | 3.89±1.079 | 4 (2) |
| **8** | “Substitution of brand-name with generic medicines can also be done by pharmacists” | 0.942 | 3.54±1.348 | 4 (3) |
| **9** | “Substitution of brand-name with generic medicines should only be done by doctors” | 0.924 | 4.23±1.080 | 5 (1) |

^a^ IQR, Interquartile range
^b^ Cronbach’s alpha for summary scale
