# Supplementary material for: Development and validation of a tool to assess knowledge and attitudes towards generic medicines among students in Greece: The ATtitude TOwards GENerics (ATTOGEN) questionnaire
Source: PLoS One. 2017 Nov 29;12(11):e0188484. doi: 10.1371/journal.pone.0188484 (PMC5706728; doi:10.1371/journal.pone.0188484)
Supplement: S7 Table — (DOCX) [file pone.0188484.s011.docx]

**Table 7. Multi-trait analysis with polychoric correlations^a^.**

| Scale/item | Item-Scale Correlations | | | | | |
| --- | --- | --- | --- | --- | --- | --- |
|  | **Trust** | **Drug quality** | **State audit** | **Fiscal impact** | **Knowledge** | **Drug substitution** |
| ***Trust*** |  |  |  |  |  |  |
| **12** | 0.671 | -0.516 | -0.227 | -0.279 | -0.116 | -0.199 |
| **13** | 0.626 | -0.384 | -0.176 | -0.272 | -0.174 | -0.156 |
| **16** | 0.661 | -0.533 | -0.198 | -0.313 | -0.226 | -0.182 |
| **20** | 0.743 | -0.591 | -0.266 | -0.307 | -0.263 | -0.216 |
| ***Drug quality*** |  |  |  |  |  |  |
| **4** | -0.481 | 0.634 | 0.172 | 0.267 | 0.352 | 0.184 |
| **5** | -0.611 | 0.753 | 0.374 | 0.344 | 0.356 | 0.219 |
| **6** | -0.500 | 0.711 | 0.419 | 0.392 | 0.274 | 0.186 |
| ***State audit*** |  |  |  |  |  |  |
| **17** | -0.283 | 0.365 | 0.864 | 0.363 | 0.086 | 0.191 |
| **18** | -0.267 | 0.356 | 0.870 | 0.336 | 0.062 | 0.146 |
| **19** | -0.168 | 0.266 | 0.632 | 0.297 | 0.075 | 0.130 |
| ***Fiscal impact*** |  |  |  |  |  |  |
| **7** | -0.191 | 0.284 | 0.291 | 0.443 | 0.124 | 0.100 |
| **10** | -0.313 | 0.310 | 0.292 | 0.520 | 0.005 | 0.269 |
| **11** | -0.353 | 0.347 | 0.305 | 0.623 | 0.168 | 0.202 |
| ***Knowledge*** |  |  |  |  |  |  |
| **1** | -0.184 | 0.256 | 0.067 | 0.039 | 0.771 | -0.003 |
| **2** | -0.178 | 0.274 | 0.064 | -0.011 | 0.822 | -0.001 |
| **3** | -0.305 | 0.481 | 0.066 | 0.141 | 0.612 | 0.046 |
| ***Drug substitution*** |  |  |  |  |  |  |
| **8** | -0.153 | 0.208 | 0.198 | 0.252 | 0.061 | 0.680 |
| **9** | -0.283 | 0.238 | 0.090 | 0.199 | 0.041 | 0.680 |

^a^ Pearson and Spearman correlations were also calculated, with similar outcomes (results not shown)
